# Efficacy of combination scalp acupuncture for post-stroke cognitive impairment: a systematic review and meta-analysis

**DOI:** 10.3389/fnins.2024.1468331

**Published:** 2024-11-29

**Authors:** Song Li, Anhong Dai, Yihao Zhou, Xu Chen, Yizhou Chen, Li Zhou, Xiaolin Yang, Mengqi Yue, Jing Shi, Yong Qiu

**Affiliations:** ^1^Yunnan University of Traditional Chinese Medicine, Kunming, China; ^2^Department of Acupuncture and Moxibustion, Yunnan Provincial Hospital of Traditional, Chinese Medicine, The First Affiliated Hospital of Yunnan University of Chinese Medicine, Kunming, China; ^3^Yan’an Hospital Affiliated to Kunming Medical University, Kunming, China; ^4^Second Clinical Medical College, Heilongjiang University of Chinese Medicine, Harbin, China

**Keywords:** scalp acupuncture, stroke, cognitive impairment, meta-analysis, randomized controlled trials

## Abstract

**Objective:**

This systematic review and meta-analysis aimed to evaluate the efficacy of combination scalp acupuncture in treating post-stroke cognitive impairment.

**Methods:**

A comprehensive search was conducted across eight databases: PubMed, Web of Science, Cochrane Database, Embase, CBM, CNKI, WanFang, and VIP, targeting randomized controlled trials (RCTs) published from the inception of these databases until October 24, 2024. The inclusion criteria focused on RCTs that compared scalp acupuncture with conventional treatments as therapeutic interventions for patients suffering from post-stroke cognitive impairment (PSCI). The effectiveness of these treatments was evaluated using various outcome measures, including the Mini-Mental State Examination (MMSE), the Montreal Cognitive Assessment (MoCA), the Loewenstein Occupational Therapy Cognitive Assessment (LOTCA), as well as P300 latency and amplitude, which collectively assess cognitive function. Two independent reviewers conducted a risk of bias (ROB2) assessment, and data analysis was performed using Review Manager (RevMan) version 5.4.

**Results:**

This analysis included a total of 28 studies involving 1,995 patients. However, according to the standards of the ROB2 tool, most of these studies exhibited various methodological issues. The comprehensive analysis indicates that the efficacy of combined scalp acupuncture in treating post-stroke cognitive impairment (PSCI) is superior to that of single treatments, as evidenced by improvements across multiple scales, including the Montreal Cognitive Assessment (MoCA), Mini-Mental State Examination (MMSE), Loewenstein Occupational Therapy Cognitive Assessment (LOTCA), P300 latency, and amplitude. Specifically, the overall effective rate was reported as (RR = 1.28, 95% CI: 1.14–1.45, *p* < 0.0001; *I*^2^ = 51%, random effects model). The mean differences for the various scales were as follows: MoCA (MD = 3.55, 95% CI: 2.68–4.41, *I*^2^ = 93%, random effects model), MMSE (MD = 3.78, 95% CI: 2.83–4.73, *I*^2^ = 94%, random effects model), LOTCA (MD = 9.70, 95% CI: 7.72–11.69, *I*^2^ = 57%, random effects model), P300 latency (MD = −21.83, 95% CI: −26.31 to −17.35, *I*^2^ = 55%, random effects model), and amplitude (MD = 1.05, 95% CI: 0.76–1.34, *I*^2^ = 0%, fixed effects model), demonstrating low, medium, and high levels of heterogeneity, respectively. Notably, one study reported an adverse event related to participant withdrawal during the study.

**Conclusion:**

Combination scalp acupuncture exhibits superior efficacy compared to single-treatment modalities in patients with post-stroke cognitive impairment (PSCI). However, the higher risk of bias (ROB) in the included trials suggests that the quality of evidence about these assessment results may be compromised. Therefore, there is an urgent need for additional high-quality clinical trials to further validate the efficacy and effectiveness of combined scalp acupuncture in treating PSCI, ultimately enhancing the overall level of evidence.

**Systematic review registration:**

https://www.crd.york.ac.uk/prospero/, identifier CRD42024519200.

## 1 Introduction

Stroke is a predominant cause of mortality and disability globally. A 2019 survey conducted by the World Health Organization (WHO) identifies stroke as the second leading cause of death, responsible for 11% of total fatalities, with a significant proportion of survivors experiencing various functional disabilities (Feigin et al., 2022). Post-stroke cognitive impairment (PSCI) encompasses a broad spectrum of neurological disorders characterized by varying degrees of cognitive dysfunction that may persist for 3 to 6 months following a stroke (Wang and Dong, 2021). The prevalence of post-stroke cognitive impairment (PSCI) ranges from approximately 17 to 66%, with variations influenced by factors such as country, race, and diagnostic criteria (Li M. et al., 2023). PSCI primarily manifests as impairments in higher-level cognitive functions, including learning, memory, executive function, and visuospatial abilities following a stroke. Common symptoms encompass attention deficits, memory impairments, and difficulties in learning (Levine et al., 2015). These cognitive challenges significantly diminish the patient’s quality of life and impose substantial financial and psychological burdens on the patient and their family. Consequently, the pursuit of a safe, effective, affordable, and acceptable treatment has become an urgent priority.

Current effective methods for enhancing cognitive function in stroke patients primarily include pharmacological treatments such as nimodipine, donepezil, and piracetam, as well as cognitive function training (CFT), the use of virtual environments and nursing care (Chang et al., 2011; Rao et al., 2013; Li Y. et al., 2023). Furthermore, research has demonstrated that several other neuroprotective agents can safely and effectively ameliorate cognitive impairment to some degree (Zhang et al., 2014). Nevertheless, long-term treatment presents several challenges that warrant attention, including potential liver and kidney toxicity, gastrointestinal issues, high costs, and concerns regarding patient compliance (Marucci et al., 2021).

Acupuncture has been widely employed in the treatment of stroke patients, addressing cognitive impairment (Liu Y. et al., 2023), dysphagia (Zhu et al., 2023), and motor functions (Bao et al., 2021). Systematic reviews and meta-analyses suggest that acupuncture may enhance post-stroke cognitive impairment (PSCI); however, the considerable heterogeneity among studies raises concerns regarding the reliability of these findings (Wu et al., 2024). Scalp acupuncture (SA), a novel approach distinct from traditional acupuncture, targets specific reflex areas of the brain that correspond to anatomical structures on the surface of the head. This technique stimulates particular regions of the scalp, which can exert direct or indirect effects on the associated brain functional areas, thereby improving cerebral blood circulation and regulating neurotransmitter release, ultimately facilitating neurological recovery. Research has also confirmed its efficacy in ameliorating the condition of patients with PSCI (Du et al., 2018; Chen J. et al., 2020; Xiong et al., 2020). Furthermore, studies suggest that the mechanisms by which acupuncture enhances PSCI involve the inhibition of nuclear factor (NF)-κB and its downstream target gene P53, as well as the modulation of neuronal apoptosis and hippocampal synaptic plasticity (Feng et al., 2013; Yang J. W. et al., 2019; Zhang et al., 2023). Additionally, some research indicates that electroacupuncture can reduce the expression and activity of calmodulin (CaM) while simultaneously increasing calmodulin-dependent protein kinase type IV (CaMKIV) and cyclic adenosine monophosphate response element-binding protein (CREB), along with their related phosphorylation, thereby improving cognitive function (Zhang et al., 2016).

Current research is deficient in data analysis concerning the efficacy of scalp acupuncture treatment for PSCI, which limits the availability of credible evidence. To scientifically assess the effectiveness of scalp acupuncture in treating PSCI, this study conducts a comprehensive analysis of randomized controlled trial data from the establishment of the database up to October 24, 2024. By evaluating multiple outcome indicators, this research aims to provide more robust evidence to support clinical practice.

## 2 Methods

### 2.1 Protocol and registration

This study was conducted as a meta-analysis by the Cochrane Handbook for Systematic Reviews of Interventions and has been registered with PROSPERO under registration number CRD 42024519200. Our research strictly adhered to the Preferred Reporting Items for Systematic Reviews and Meta-Analyses (PRISMA) reporting guidelines (Page et al., 2021). The PRISMA checklist is provided in the Appendix 1.

### 2.2 Literature search

We conducted a comprehensive search across eight databases, which included four English databases—PubMed, Cochrane Library, Embase, and Web of Science (WoS)—and four Chinese databases—China Biomedicine (CBM), China National Knowledge Infrastructure (CNKI), Chinese Science and Technology Journals (VIP), and WanFang Database. The search covered the period from the establishment of each database up to October 24, 2024, without restrictions on country, language, or publication status. We utilized the MeSH subject headings “scalp acupuncture,” “stroke,” and “cognitive impairment,” with a focus on randomized controlled trials (RCTs) to develop our search strategy. This strategy was specifically tailored to accommodate the unique characteristics of each database. Detailed search terms and strategies for each database are provided in Appendix 2. Additionally, we manually screened the reference lists of all included articles to identify potentially relevant RCTs.

### 2.3 Inclusion and exclusion criteria

The studies included in this analysis were not restricted by age, gender, race, or ethnicity. The inclusion is as follows: (1) research subjects must meet the diagnostic criteria for stroke patients; (2) the intervention involves scalp acupuncture; (3) the control group consists of sham acupuncture or other treatment methods such as primary treatment or blank control; (4) the study design must be a RCTs; (5) the research must include complete data recording and analysis.

The exclusion criteria are (1) non-randomized controlled trials; (2) exclusion of animal studies; (3) dissertations, conference papers, and case reports; (4) unavailability of original text or full-text data; (5) review articles.

### 2.4 Study selection and data extraction

Two researchers independently screened the literature included in this study based on established inclusion and exclusion criteria. They selected the titles and abstracts of relevant literature to identify qualified studies for retention. A third researcher assessed any controversial literature. Basic information from the included studies was extracted, including the first author’s name, year of publication, subject status, sample size, intervention measures, and outcome indicators. For articles with incomplete data, the original authors were contacted via email or telephone to obtain any missing or unclear information.

### 2.5 Risk of bias in individual studies

All included studies were RCTs, and the Cochrane Risk of Bias Tool 2.0 (RoB 2) was utilized to assess the risk of bias within these trials. This comprehensive tool evaluates potential biases across five key domains: (1) the randomization process, (2) deviations from the intended intervention, (3) instances of missing outcome data, (4) methods of outcome measurement, and (5) criteria for the selection of reported outcomes. Each domain is classified according to the risk of bias as low risk, high risk, or some concern. To maintain rigorous methodological standards, the risk of bias for each included study was independently assessed by two researchers. A third researcher addressed any discrepancies or inconsistencies identified during the independent assessment to ensure an unbiased evaluation.

### 2.6 Data analysis

This study utilized RevMan 5.4 software to perform a meta-analysis. The mean difference (MD) is reported as the effect size for continuous outcome measures. In the case of binary variables, the relative risk (RR) and the corresponding 95% confidence interval (CI) represent the effect size. We employed the *χ*^2^ test with *I*^2^ quantitative analysis to assess inter-study heterogeneity. If *p* > 0.1 and *I*^2^ < 50%, it is deemed that there is no significant heterogeneity among the included studies, and a fixed effects model is applied for the meta-analysis. Conversely, if *p* < 0.1 and *I*^2^ > 50%, it indicates a significant heterogeneity among the studies. Lastly, we conducted subgroup and sensitivity analyses to investigate the sources of heterogeneity and gain a deeper understanding of the intervention’s effects. Publication bias was evaluated using funnel plots and Egger’s bias test.

## 3 Results

### 3.1 Study selection

**Figure 1:**
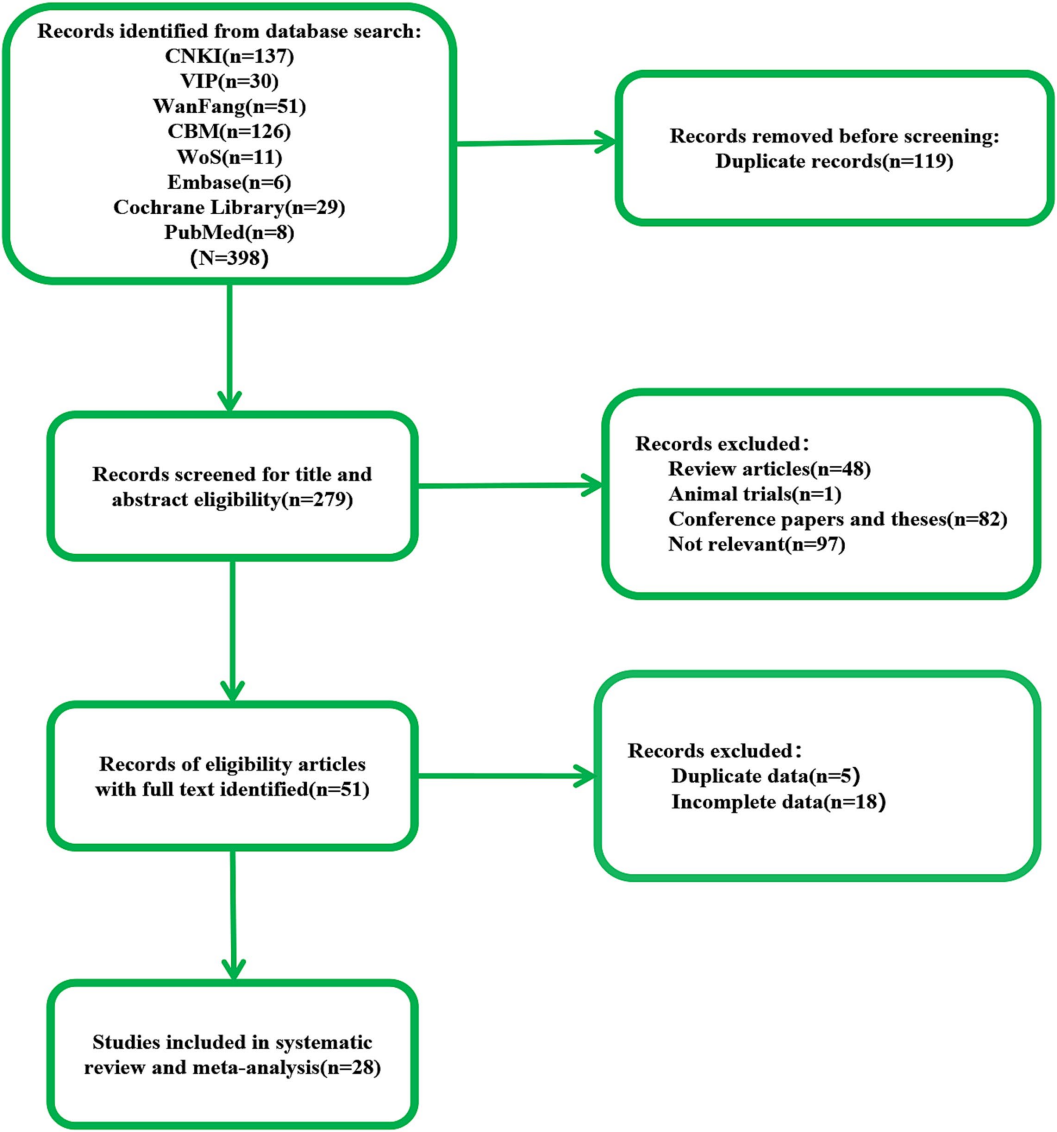
The flow of literature search and selection.

According to the search strategy, we retrieved 398 documents. Our preliminary screening excluded 119 duplicate documents, 82 conference papers and theses, and 49 reviews, systematic reviews, meta-analyses, and animal experiments. Reviewing the titles, abstracts, and keywords, we identified 97 documents that did not align with the research content. Upon examining the complete texts, we discovered that 23 articles contained duplicate or incomplete data. Ultimately, 28 articles were included in this study (Figure 1).

### 3.2 Study characteristics

All studies included in this analysis were RCTs. A total of 28 studies involving 1995 participants were examined, with 998 in the treatment group and 997 in the control group. Among the included studies, four were conducted in English (Xie et al., 2012; Du et al., 2018; Chen J. et al., 2020; Xiong et al., 2020), while the remaining 24 were conducted in Chinese (Xie et al., 2007; Fu et al., 2010; Wang et al., 2012; Qin, 2015; Ding and Zhang, 2016; Wang, 2016; Huang et al., 2017; Wang et al., 2017; Han et al., 2018; Wang W. et al., 2018; Wang Y. X. et al., 2018; Zhang and An, 2018; Yang L. et al., 2019; Liu et al., 2019; Mao and Cheng, 2019; Zhang et al., 2019; Chen A. Z. et al., 2020; Zhang et al., 2020; Cai et al., 2022; Zhai et al., 2022; Li and Pan, 2023; Wei et al., 2023; Liu Y. F. et al., 2023; Liu et al., 2024), all within China. There was no statistically significant difference in the baseline characteristics of the treatment and control groups regarding age, disease duration, education level, outcome indicators, and other basic information before enrollment. The intervention methods varied across studies. One study exclusively utilized scalp acupuncture combined with basic treatment (B) (Wang et al., 2012), while the other 27 studies incorporated additional intervention methods, including rehabilitation therapy (R) (Xie et al., 2007; Fu et al., 2010; Xie et al., 2012; Qin, 2015; Ding and Zhang, 2016; Huang et al., 2017; Wang W. et al., 2018; Wang Y. X. et al., 2018; Mao and Cheng, 2019; Zhang et al., 2019; Cai et al., 2022; Wei et al., 2023; Liu et al., 2024), computer-assisted cognitive training (CACT) (Wang, 2016; Zhang et al., 2020) hyperbaric oxygen therapy (H) (Wang et al., 2017; Yang L. et al., 2019), repetitive transcranial magnetic stimulation (rTMS) (Han et al., 2018; Liu et al., 2019), cognitive training (CT) (Du et al., 2018; Chen A. Z. et al., 2020; Xiong et al., 2020; Zhai et al., 2022; Li and Pan, 2023), the donepezil hydrochloride (Chen J. et al., 2020; Liu Y. et al., 2023) and the drug idebenone (Zhang and An, 2018). The control group interventions included nimodipine, CT, donepezil hydrochloride, R, CACT, H, rTMS, idebenone, and conventional symptomatic treatment.

The 28 studies included in this analysis employed four evaluation tools—MoCA, MMSE, LOTCA, and P300—as outcome indicators for scalp acupuncture treatment of post-stroke cognitive impairment (PSCI). Among these, 13 studies (Wang et al., 2017; Wang W. et al., 2018; Yang L. et al., 2019; Liu et al., 2019; Mao and Cheng, 2019; Chen A. Z. et al., 2020; Chen J. et al., 2020; Zhang et al., 2020; Cai et al., 2022; Li and Pan, 2023; Wei et al., 2023; Liu Y. F. et al., 2023; Liu et al., 2024) utilized the MoCA scale as their primary outcome measure. In contrast, 14 studies (Ding and Zhang, 2016; Han et al., 2018; Wang W. et al., 2018; Wang Y. X. et al., 2018; Zhang and An, 2018; Yang L. et al., 2019; Liu et al., 2019; Zhang et al., 2019; Xiong et al., 2020; Cai et al., 2022; Zhai et al., 2022; Wei et al., 2023; Liu Y. et al., 2023; Liu et al., 2024) selected the MMSE scale as their outcome measure, while seven studies (Wang W. et al., 2018; Yang L. et al., 2019; Liu et al., 2019; Cai et al., 2022; Wei et al., 2023; Liu Y. F. et al., 2023; Liu et al., 2024) employed both the MoCA and MMSE scales as outcome measures. Additionally, six studies (Fu et al., 2010; Wang et al., 2012; Wang, 2016; Huang et al., 2017; Du et al., 2018; Xiong et al., 2020) utilized the LOTCA score as an outcome measure. Among the 28 studies, seven studies (Xie et al., 2012; Zhang and An, 2018; Yang L. et al., 2019; Mao and Cheng, 2019; Zhai et al., 2022; Wei et al., 2023; Liu Y. F. et al., 2023) indicated total effectiveness as an outcome indicator. Furthermore, five studies (Xie et al., 2007; Xie et al., 2012; Qin, 2015; Wang Y. X. et al., 2018; Wei et al., 2023) assessed P300’s latency and amplitude as evaluation tools. The included studies primarily employed the Barthel index (BI) and modified Barthel index (MBI) scores to evaluate daily activities. Notably, one study reported an adverse event involving participant withdrawal during the investigation. The characteristics of all 28 studies are summarized in Table 1.

**Table 1:** Characteristics of included studies.

Study	Year	Gender		Age		Random method	Cases		Intervention		Outcome measures
		Male	Female	TG	CG		TG	CG	TG	CG	
Liu	2024	26	34	55.7 ± 13.22	60.0 ± 10.09	Random number table method	30	30	SA + R	R	MoCA, MMSE, MBI
Wang	2016	28	12	61.8 ± 11.92	60.25 ± 10.39	Not mentioned	20	20	SA + CACT	CACT	LOTCA, MBI, FMA
Zhang	2018	50	26	69.5 ± 5.5	68.9 ± 4.9	Random number table method	38	38	SA + Idebenone	Idebenone	MMSE, HAMD-24, BI, SF-36, effective rate
Li	2023	61	19	68.6 ± 2.5	67.9 ± 3.7	Random number table method	40	40	SA + CT	CT	MoCA, NIHSS, BI, ET-1
Wang	2012	/	/	56.1 ± 8.6		Random number table method	25	25	SA + B	B	LOTCA
Xie	2007	46	34	53.0 ± 9.3	56.5 ± 6.4	Not mentioned	41	39	SA + R	R	P300, FMA, BI, ADL
Qin	2015	75	41	64.7 ± 5.66	65.3 ± 7.87	Random number table method	58	58	SA + R	R	FMA, P300, QLQ-C30
Liu	2019	41	39	55.00 ± 13.84	54.31 ± 13.31	Not mentioned	41	39	SA + rTMS	rTMS	MoCA, MMSE
Zhai	2022	49	41	60.22 ± 1.20	60.39 ± 7.23	Random number table method	45	45	SA + CT	CT	MMSE, ADL, effective rate
Han	2018	22	8	60.3 ± 8.1	61.5 ± 8.0	Random number table method	15	15	SA + rTMS	rTMS	MMSE, BI
Fu	2010	37	47	57.2 ± 5.2		Randomized in order of visited	42	42	SA + R	R	LOTCA, QOL
Huang	2017	39	25	57.2 ± 2.8	57.4 ± 3.2	Randomized in order of visited	32	32	SA + R	R	BI, LOTCA
Wang A	2018	49	31	45.39 ± 11.42	42.29 ± 11.72	Random number table method	40	40	SA + R	R	MMSE, P300, Hcy
Yang	2019	31	29	52.14 ± 7.56	51.21 ± 7.84	Random number table method	30	30	SA + H	H	MMSE, MoCA, effective rate
Zhang	2020	38	22	70.10 ± 4.51	69.03 ± 4.70	Random number table method	30	30	SA + CACT	CACT	MoCA, NAA/Cr, Cho/Cr, MI/Cr
Mao	2019	37	41	57.3 ± 15.6	58.2 ± 16.2	Not mentioned	39	39	SA + R	R	effective rate, MoCA, MBI
Wang	2017	/	/	69.5 ± 4.25	67.6 ± 3.67	Not mentioned	19	18	SA + H	H	MOCA, BI
Zhang	2019	35	33	64.3 ± 9.2	64.0 ± 9.6	Not mentioned	34	34	SA + R	R	FMA, MMSE, MBI
Liu	2023	37	26	62 ± 5	62 ± 4	Random number table method	32	31	SA + donepezil hydrochloride	Donepezil hydrochloride	MMSE, MoCA, ADL, SS-QOL, effective rate
Ding	2016	48	38	56.24 ± 8.12	57.87 ± 9.01	Not mentioned	40	46	SA + R	R	MMSE, BI
Wei	2023	52	28	53.18 ± 8.42	52.31 ± 7.87	Random number table method	40	40	SA + R	R	effective rate, MMSE, MoCA, MBI, P300
Chen	2020	27	33	62.00 ± 5.12	61.77 ± 4.81	Not mentioned	30	30	SA + CT	CT	MoCA, MBI
Wang B	2018	50	50	53.8 ± 11.7	54.5 ± 13.6	Not mentioned	50	50	SA + R	R	MoCA, MMSE, ADL
Cai	2022	77	43	54.82 ± 13.24	55.62 ± 13.08	Random number table method	60	60	SA + R	R	MMSE, MoCA, VEGF, NO, ET-1
Xie	2012	44	22	/		Not mentioned	34	32	SA + R	R	P300, effective rate
Jin	2018	39	21	40.63 ± 5.68	37.00 ± 7.25	Random number table method	30	30	SA + CT	CT	LOTCA
Jian	2020	37	33	63.0 ± 7.23	65.3 ± 8.52	Random number table method	35	35	SA + CT	CT	MMSE, LOTCA, FMA, BDNF, NGF
Jing	2020	33	23	64.5 ± 7.2	65.3 ± 6.8	Not mentioned	28	28	SA + donepezil hydrochloride	Donepezil hydrochloride	MoCA, ADL, cerebraloxy-Hblevel, cerebraldeoxy-Hblevel, cerebraltotalHblevel

TG, therapy group; CG, control group; SA, scalp acupuncture; CACT, computer-assisted cognitive training; CT, cognitive training; R, rehabilitation; B, basic treatment; H, hyperbaric oxygen therapy; rTMS, repetitive transcranial magnetic stimulation; Hcy, homocysteine; MoCA, Montreal Cognitive Assessment; MMSE, Minimum Mental State Examination; BI, the Barthel Index Rating Scale for activities of daily living; LOTCA, Loewenstein Occupational Therapy Cognitive Assessment; MBI, the Modified Barthel Index Rating Scale for activities of daily living; FMA, Fugl-Meyer Assessment; HAMD-24, Hamilton Depression Scale; SF-36, the MOS item short from health survey; NIHSS, NIHSS score on the National Institutes of Health Stroke Scale; ADL, activities of daily living; SS-QOL, Stroke Specific Quality of Life Scale; VEGF, vascular endothelial growth factor; ET-1, endothelin-1; QLQ-C30, The Quality of Life Questionnaire Core 30; BDNF, brain-derived neurotrophic factor; NGF, nerve growth factor.

The characteristics of SA parameters vary across the 28 studies examined. Regarding SA type, 19 studies employed International SA (Xie et al., 2007; Fu et al., 2010; Wang et al., 2012; Xie et al., 2012; Qin, 2015; Ding and Zhang, 2016; Wang, 2016; Huang et al., 2017; Wang et al., 2017; Du et al., 2018; Wang W. et al., 2018; Yang L. et al., 2019; Liu et al., 2019; Mao and Cheng, 2019; Zhang et al., 2019; Zhang et al., 2020; Cai et al., 2022; Liu et al., 2024), while two studies utilized Fang SA (Zhang and An, 2018; Liu Y. F. et al., 2023), and another two studies employed Yu SA (Wang Y. X. et al., 2018; Wei et al., 2023). Additionally, 1 study focused on Lingnan SA (Li and Pan, 2023). In terms of treatment duration, 17 studies administered sessions lasting 30 min each (Xie et al., 2007; Wang et al., 2012; Qin, 2015; Wang, 2016; Wang et al., 2017; Du et al., 2018; Han et al., 2018; Wang W. et al., 2018; Zhang and An, 2018; Yang L. et al., 2019; Liu et al., 2019; Chen A. Z. et al., 2020; Zhang et al., 2020; Cai et al., 2022; Li and Pan, 2023; Liu Y. F. et al., 2023; Liu et al., 2024), whereas 1 study lasted 40 min (Ding and Zhang, 2016), another lasted 60 min (Wei et al., 2023), and 1 study had a duration of 120 min (Wang et al., 2012), furthermore, five studies reported sessions lasting 360 min each (Fu et al., 2010; Huang et al., 2017; Wang Y. X. et al., 2018; Mao and Cheng, 2019; Chen J. et al., 2020), while 1 study varied between 15 to 30 min (Zhai et al., 2022), and another ranged from 180 to 240 min (Xiong et al., 2020). The SA parameter characteristics are detailed in Table 2.

**Table 2:** The SA parameter characteristics.

Study	Country	Disease course		Stroke types		Intervention protocols					Medication
		CG	TG	Hemorrhagic stroke	Ischemic stroke	SA types	SA acupoints	Frequency (next/min)	Time (minute)	Intervention time	
Liu 2024	China	≤6 month; ≧3 weeks		✓	✓	International SA	DU20, MS6, GV24, GB13	200	30	4	—
Wang 2016	China	≤3 month		✓	✓	International SA	MS6, MS7	—	30	8	—
Zhang 2018	China	5.70 ± 2.7 0 month	6.10 ± 2.30 month	✓	✓	Fang SA	Writing point area, thinking point area, Yunping point area, balance point area, Fuxiang head point area	160	30	8	Idebenone, 30 mg/time, 3 times/day
Li 2023	China	3. 04 ± 0.75 month	2. 94 ± 0.95 month	✓	✓	Lingnan SA	GV24, ST8, GB15, GB8, TE19, GB9, TE19, GB7, TE21, GV17, GV16, BL9, BL10	—	30	4	—
Wang 2012	China	3 weeks–3 months		✓	✓	International SA	MS5, MS6, MS7	200	120	8	—
Xie 2007	China	≧3 weeks		✓	✓	International SA	MS1, MS5, MS10, MS11	200	30	12	—
Qin 2015	China	≧3 weeks		✓	✓	International SA	MS1, MS5, MS10, MS11	200	30	12	—
Liu 2019	China	7 days–6 months		—	✓	International SA	MS1, MS5, MS10	160	30	4	—
Zhai 2022	China	4.19 ± 1.15 month	4.16 ± 1.20 month	✓	✓	-	DU20, GV21, GB4	200	15–30	8	Nimodipine 30 mg/time, 3 times/day; oxiracetam, 0.8 g/time, 3 times/day
Han 2018	China	≧1 month		✓	✓	—	GV24, GB13, GV21, GV19, BL8	—	30	8	—
Fu 2010	China	≧3 weeks		✓	✓	International SA	MS6, MS7	200	360	10	—
Huang 2017	China	≧4 weeks		✓	✓	International SA	MS6, MS7	200	360	12	—
Wang A 2018	China	26.85 ± 16.10 day	24.05 ± 11.89 day	—	✓	Yu SA	DU20, GV21, DU20, GV22, GV24		360	4	—
Yang 2019	China	1–3 months		✓	✓	International SA	MS1, MS5, MS6, MS7	/	30	4	—
Zhang 2020	China	≧2 weeks		/	✓	International SA	MS1, MS6	200	30	6	—
Mao 2019	China	15 days–5 months		✓	✓	International SA	MS5, MS1, MS6, MS7, MS2, MS3	180–200	360	3	—
Wang 2017	China	1–4 weeks		—	✓	International SA	MS1, MS2, MS3, MS4	100	30	12	—
Zhang 2019	China	≤3 months		✓	✓	International SA	MS5, MS14	—	—	12	—
Liu 2023	China	4.34 ± 1.38 months	4.32 ± 1.36 months	—	✓	Fang SA	Fuxiang head area, The upper focal area of the Fu Zang, like the upper area, dirty upper focus area, signal area, writing area, memory area, thinking area	—	30	9	Donepezil hydrochloride, 5 mg/time, 3 times/day
Ding 2016	China	11.8 ± 10.41 days	14.24 ± 9.5 days	✓	✓	International SA	DU20, EX-HN1, MS6, MS7, DU20, MS10, MS11	—	40	8	—
Wei 2023	China	2.26 ± 0.93 month	2.14 ± 0.58 month	✓	✓	Yu SA	DU20, EX-HN1, GV21, GV22, BL7, BL6, GB17, GB16	200	60	4	—
Chen 2020	China	≧2 weeks		✓	✓	—	DU20, EX-HN1	200	30	4	—
Wang B 2018	China	3 weeks–3 months		✓	✓	International SA	MS6, MS7	200	30	3	—
Cai 2022	China	30.53 ± 9.54 days	32.41 ± 10.50 days	✓	✓	International SA	MS1, MS5, MS10, MS11	—	30	12	Aspirin, nimodipine
Xie 2012	China	≤3 month		—	✓	International SA	MS1, MS5, MS11	198	30	12	—
Jin 2018	China	4.70 ± 1.43 month	4.85 ± 1.27 month	✓	✓	International SA	MS5, MS1, MS10, MS11	—	30	12	Citicoline sodium 0.2 g, 3 times/day
Jian 2020	China	≧3 weeks		✓	✓	—	DU20, EX-HN1, GB20, GV24	—	180–240	12	—
Jing 2020	China	—		✓	✓	—	Parietofrontal area, temporal area, occipital area, suboccipital area, preparietal area, nuchal area	200	360	4	Donepezil hydrochloride tablets 5 mg/day

### 3.3 Study design and risk of bias

**Figure 2:**
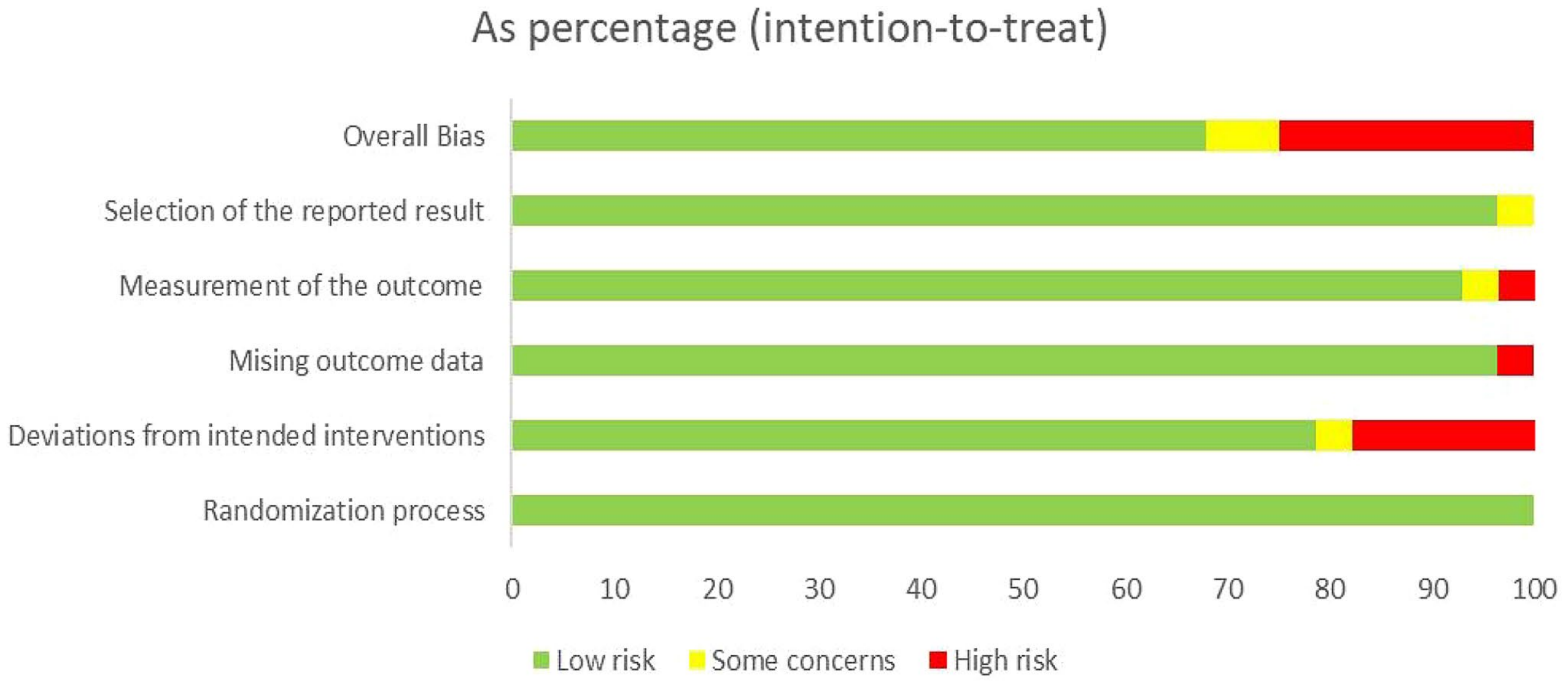
Assessment of risk of bias summary of included studies using the Cochrane tool.

**Figure 3:**
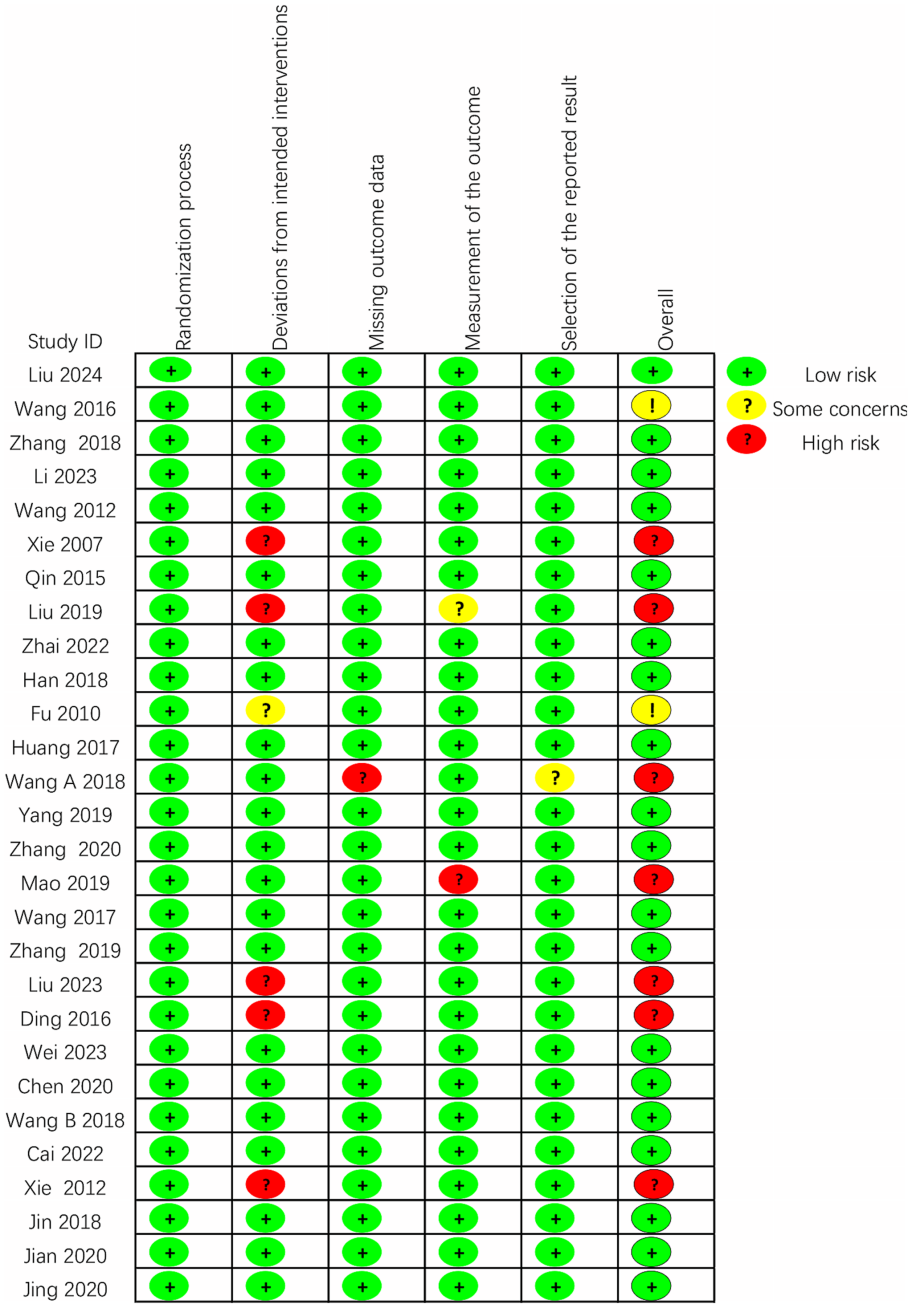
Assessment of risk of bias graph of included studies using the Cochrane tool.

The quality of the 28 included randomized controlled trials was generally classified as “low to moderate.” It is important to note that due to the unique nature of scalp acupuncture, all studies were unable to blind operators and participants. Two studies were assessed as having a risk of “some concern” (Fu et al., 2010; Wang, 2016), while 19 studies were categorized as having a low risk (Wang et al., 2012; Qin, 2015; Huang et al., 2017; Wang et al., 2017; Du et al., 2018; Han et al., 2018; Wang W. et al., 2018; Zhang and An, 2018; Yang L. et al., 2019; Zhang et al., 2019; Chen A. Z. et al., 2020; Chen J. et al., 2020; Xiong et al., 2020; Zhang et al., 2020; Cai et al., 2022; Zhai et al., 2022; Li and Pan, 2023; Wei et al., 2023; Liu et al., 2024), and seven studies were identified as having a high risk of bias (Xie et al., 2007; Xie et al., 2012; Ding and Zhang, 2016; Wang Y. X. et al., 2018; Liu et al., 2019; Mao and Cheng, 2019; Liu Y. F. et al., 2023). Regarding the randomization process, most studies were deemed to have a low risk, as the randomization methods were reported in detail, with the random number table method being the most commonly employed technique. Additionally, there was a possibility of selective reporting of results in one study (Wang W. et al., 2018). Detailed quality assessments are presented in Figures 2, 3.

### 3.4 Meta-analysis

#### 3.4.1 The total effective rate

**Figure 4:**
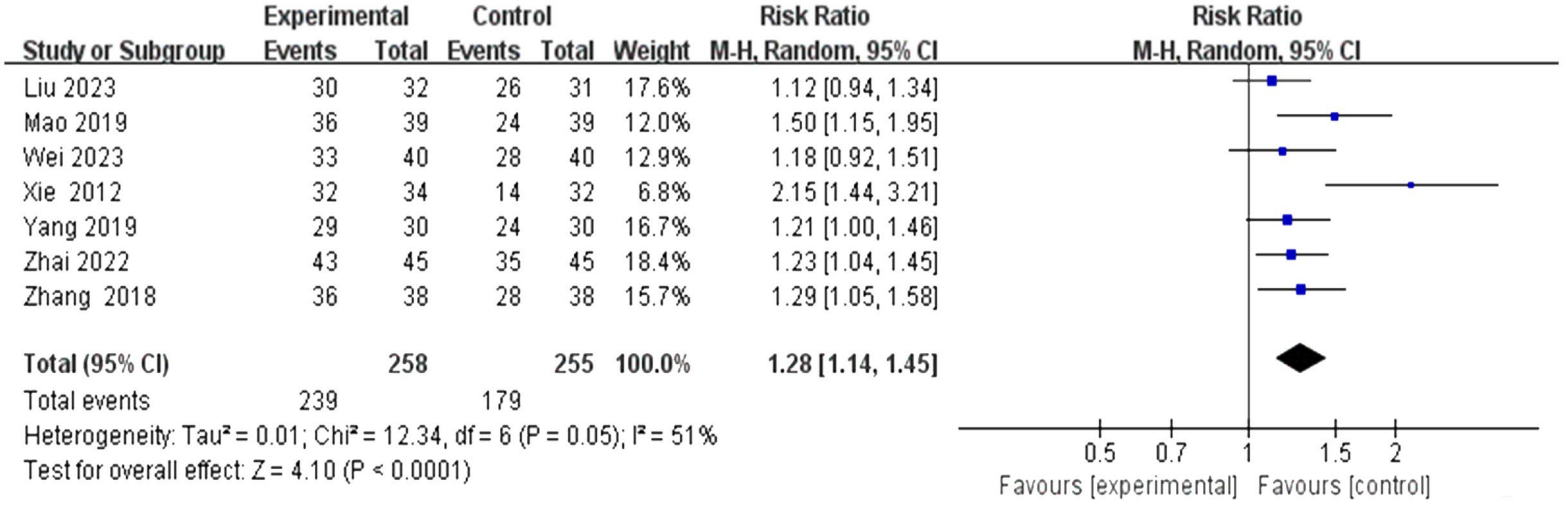
Forest plots of the total effective rate.

This study encompassed 28 individual studies, of which seven reported on this specific outcome. A subsequent meta-analysis employing a random effects model revealed that the efficacy of the combined scalp acupuncture treatment group was significantly superior to that of the control group (RR = 1.28, 95% CI: 1.14 to 1.45, *I*^2^ = 51%, 7 studies, 513 participants). Additionally, the results of the heterogeneity analysis suggest that these findings are robust (Figure 4).

#### 3.4.2 The MoCA scores

**Figure 5:**
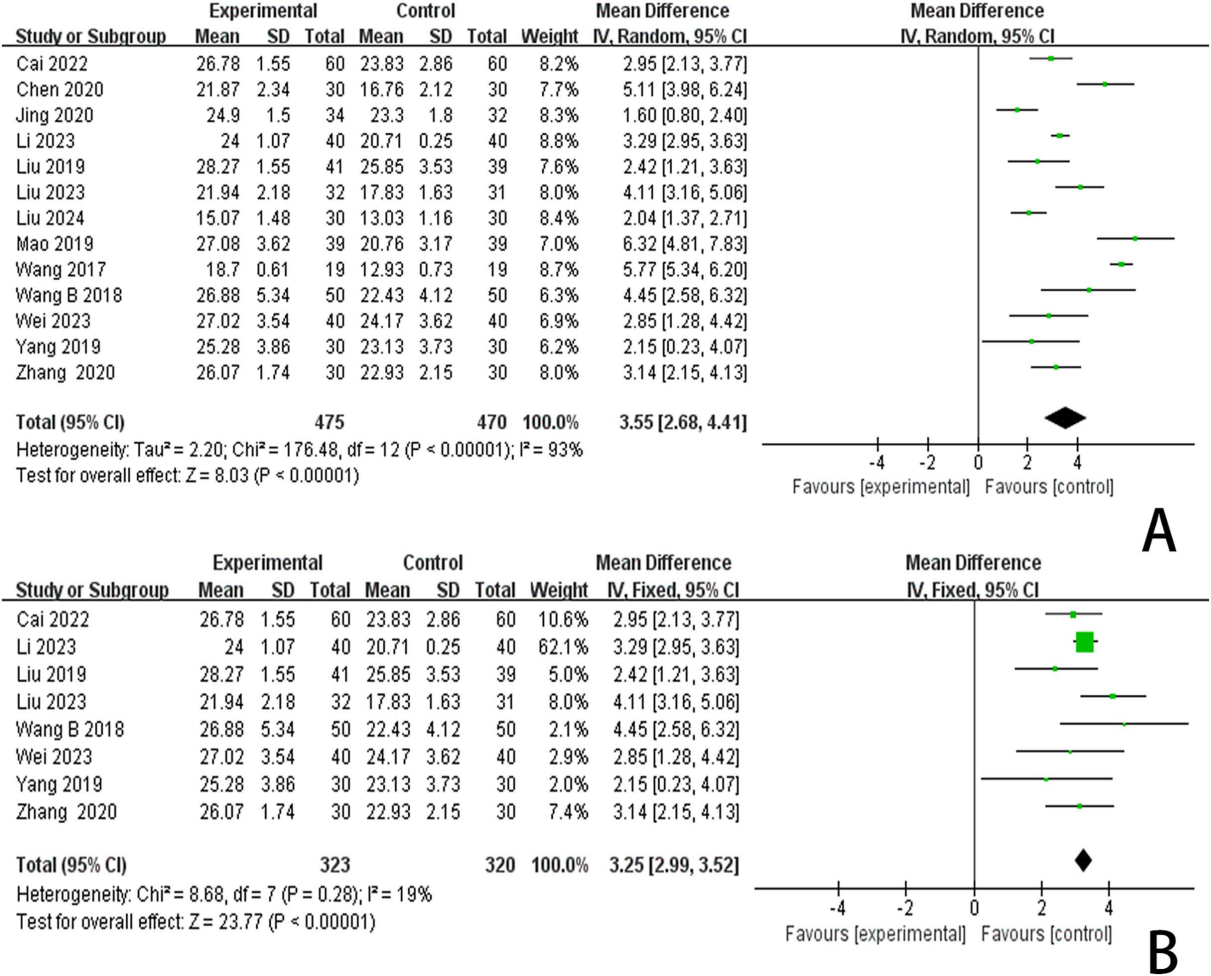
**(A)** Forest plots of the MoCA scores. **(B)** Forest plots of the MoCA scores after removing four studies.

In this study, 13 investigations reported MoCA scores. A meta-analysis of the included studies was conducted using a random effects model. The results indicated that the combination of the combined scalp acupuncture treatment was more effective than the control group in enhancing the MoCA scores of PSCI patients (MD = 3.55, 95% CI: 2.68–4.41, *I*^2^ = 93%, 13 studies, 944 participants) (Figure 5A). However, there was a high level of heterogeneity across the studies.

#### 3.4.3 The MMSE scores

**Figure 6:**
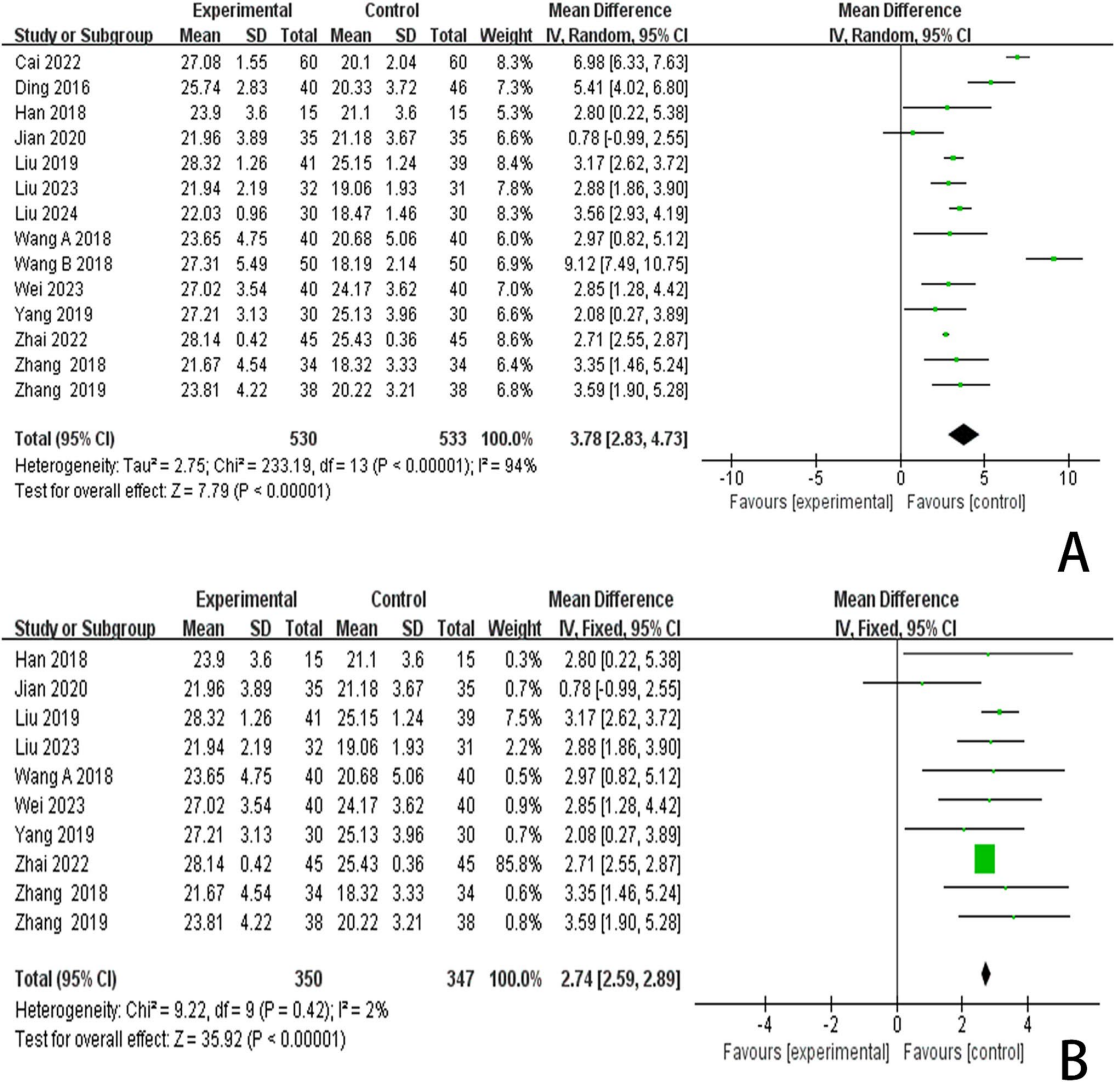
**(A)** Forest plots of the MMSE scores. **(B)** Forest plots of the MMSE scores after removing four studies.

In this study, 13 investigations reported MoCA scores. A meta-analysis of the included studies was conducted using a random effects model. The results indicated that the combination of scalp acupuncture treatment was more effective than the control group in enhancing the MoCA scores of PSCI patients (MD = 3.78, 95% CI: 2.83–4.73, *I*^2^ = 94%, 14 studies, 1,063 participants) (Figure 6A). However, there was a high level of heterogeneity across the studies.

#### 3.4.4 The LOTCA scores

**Figure 7:**
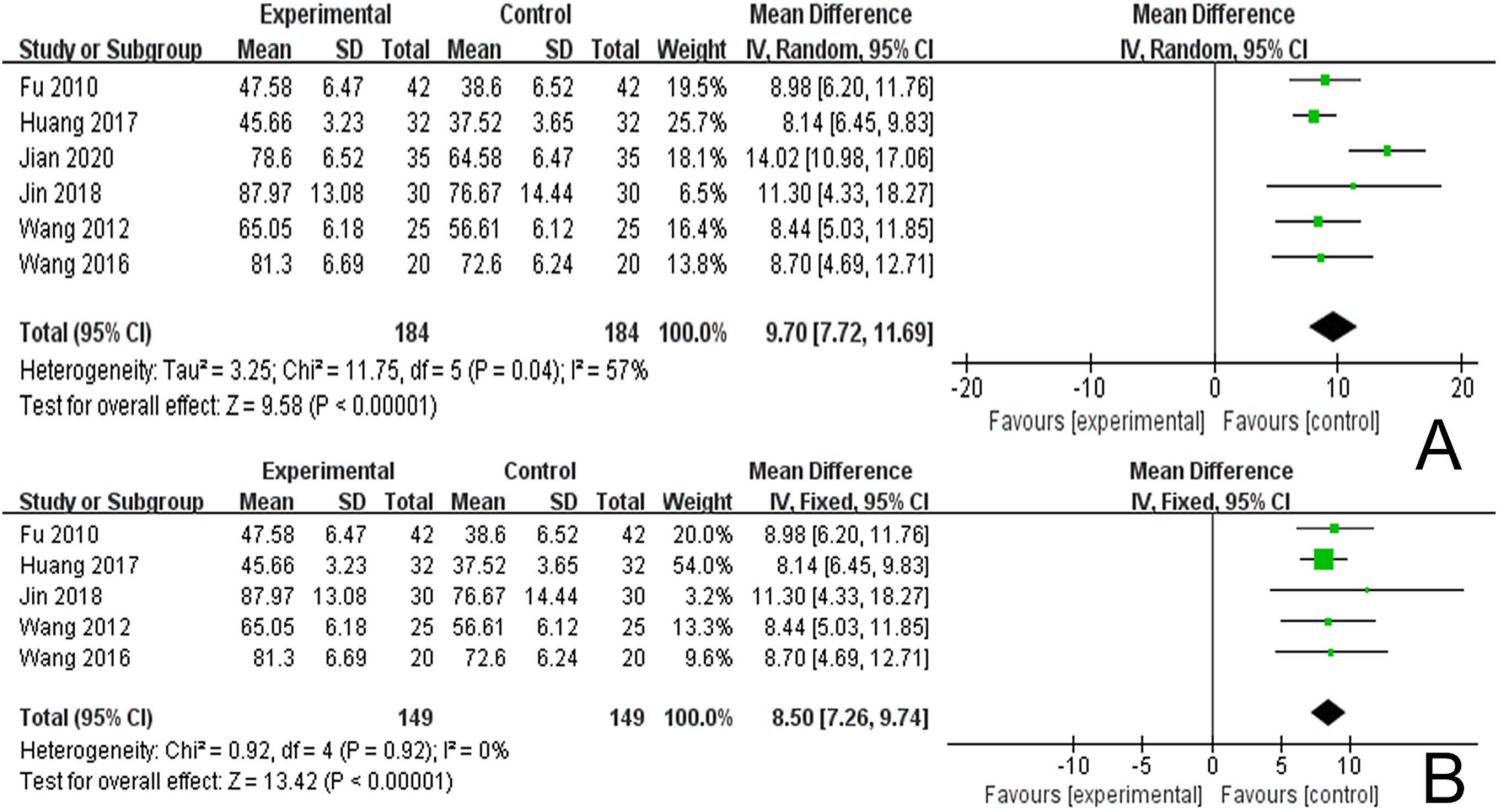
**(A)** Forest plots of the LOTCA scores. **(B)** Forest plots of the LOTCA scores after removing one study.

Six studies reported LOTCA scores and a random effects model was employed to conduct a meta-analysis of the data from each study. The results indicated that combined scalp acupuncture treatment significantly improves the LOTCA scores of PSCI patients compared to the control group (MD = 9.70, 95% CI: 7.72–11.69, *I*^2^ = 57%, 6 studies, 368 participants) (Figure 7A), with moderate heterogeneity observed between the studies.

#### 3.4.5 The P300 scores

**Figure 8:**
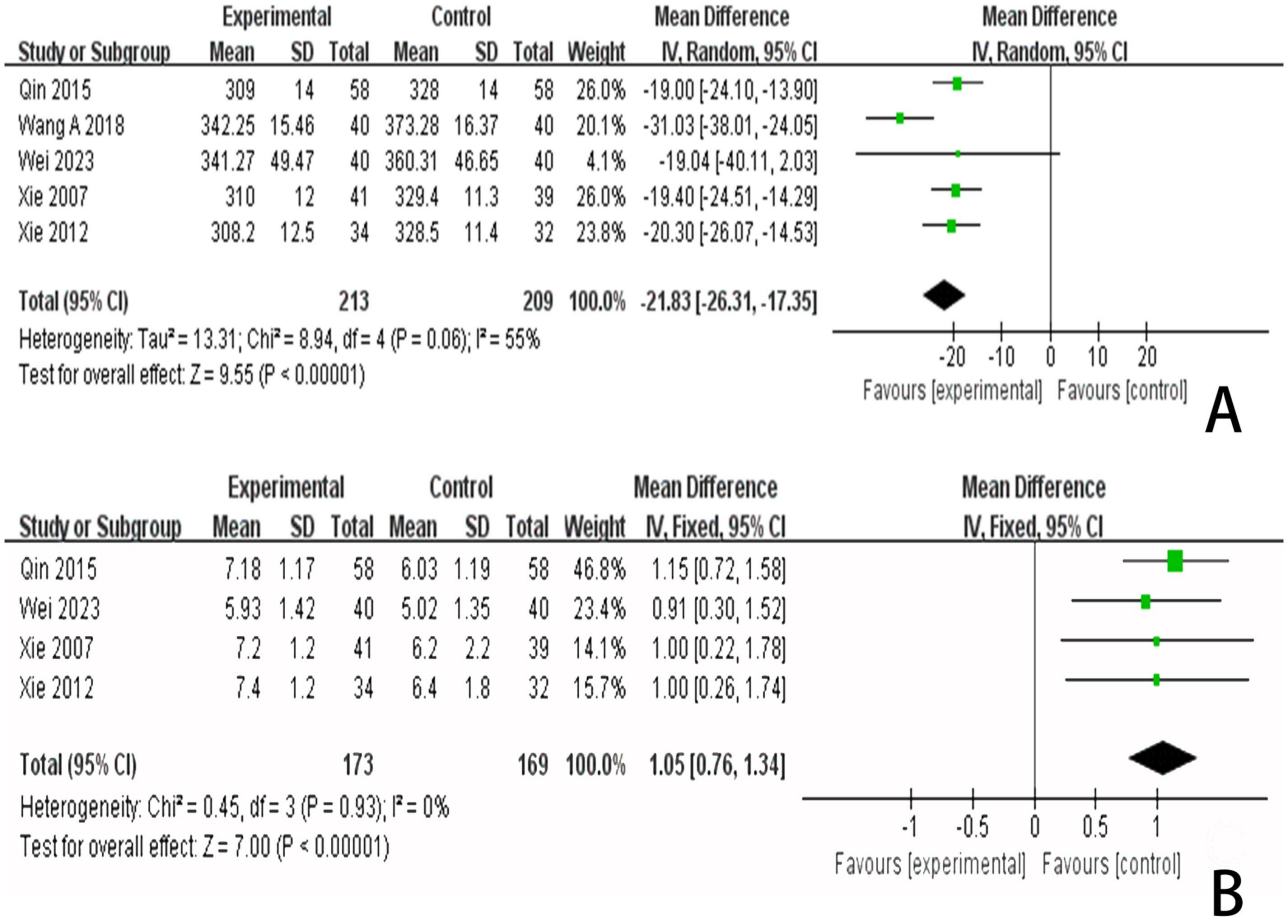
**(A)** Forest plots of the P300 latency. **(B)** Forest plots of the P300 latency after removing one study. **(C)** Forest plots of the P300 amplitude.

In reviewing the 28 studies included in this analysis, 5 reported P300 latency, while 4 studies reported P300 amplitude. We employed a random effects model to meta-analyze the latency results and found that scalp acupuncture combined with treatment significantly reduced P300 latency in patients with PSCI compared to the control group (MD = −21.83, 95% CI: −26.31 to −17.35, *I*^2^ = 55%, 5 studies, 422 participants) (Figure 8A). Additionally, a meta-analysis of the 4 studies reporting P300 amplitude, utilizing a fixed effects model, indicated that scalp acupuncture combined with treatment could enhance P300 amplitude in PSCI patients relative to the control group (MD = 1.05, 95% CI: 0.76–1.34, *I*^2^ = 0%, 4 studies, 342 participants) (Figure 8B).

### 3.5 Subgroup analysis

#### 3.5.1 Subgroup analysis of MoCA scores

**Figure 9:**
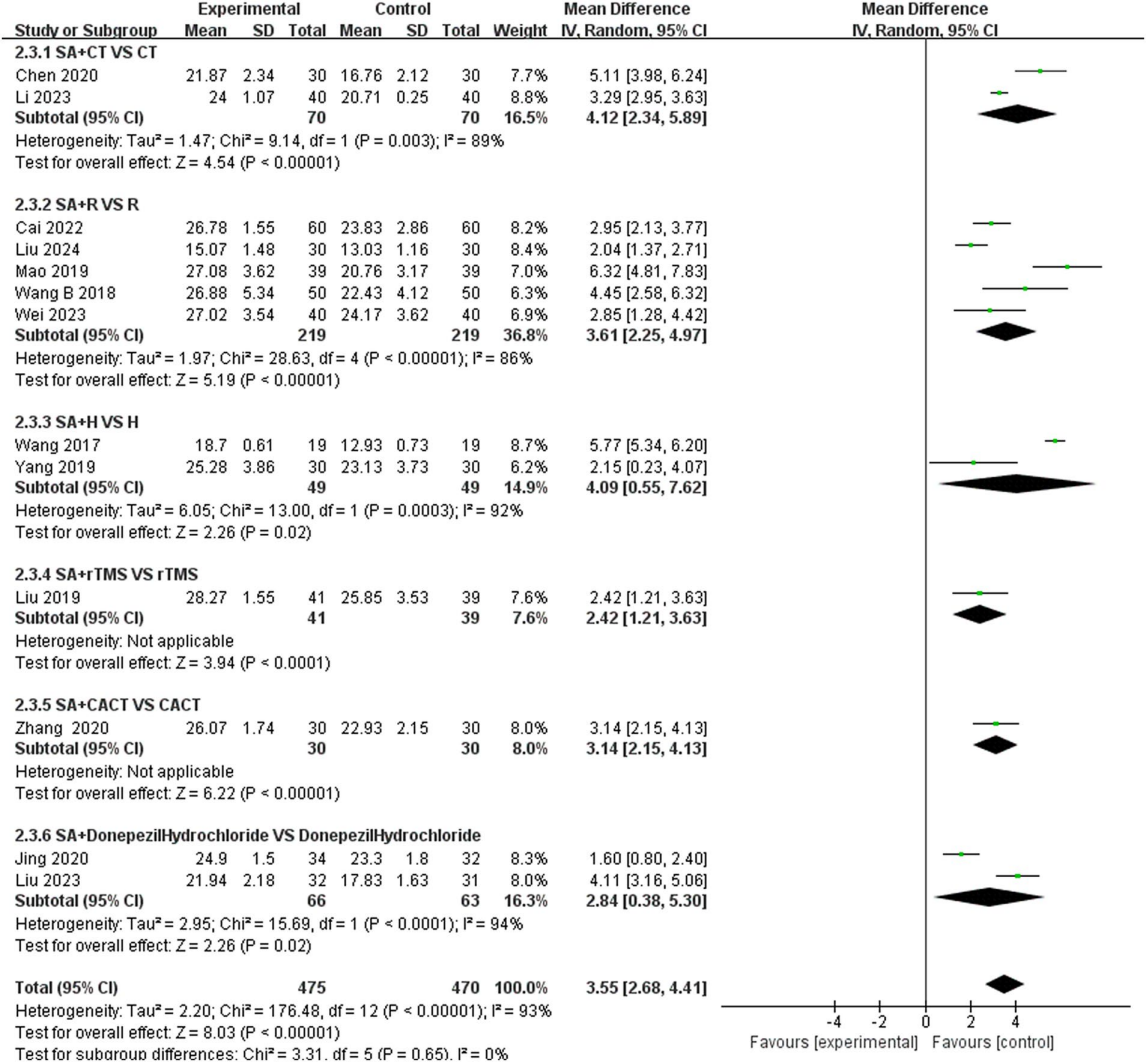
Forest plots of subgroup analysis of MoCA scores.

We conducted a subgroup analysis of studies that recorded MoCA scores based on the type of treatment administered in the control group (Figure 9). The results indicated that the SA + CT vs. CT subgroup was significantly effective (MD = 4.12, 95% CI: 2.34–5.89, *I*^2^ = 89%, 2 studies, 140 participants). Similarly, the SA + R vs. R subgroup also demonstrated significant effectiveness (MD = 3.61, 95% CI: 2.25–4.97, *I*^2^ = 86%, 5 studies, 438 participants). The SA + H vs. H subgroup showed significant effectiveness as well (MD = 4.09, 95% CI: 0.55–7.62, *I*^2^ = 92%, 2 studies, 97 participants). In the comparison of SA + donepezil hydrochloride vs. donepezil hydrochloride, the results were significant (MD = 2.84, 95% CI: 0.38–5.30, *I*^2^ = 94%, 2 studies, 129 participants). Heterogeneity was considered high, as *I*^2^ > 75% in four subgroups, indicating that the results were less reliable. Additionally, the subgroup SA + rTMS vs. rTMS showed significant results (MD = 2.42, 95% CI: 1.21–3.63, *Z* = 3.94, *p* < 0.0001), and SA + CACT vs. CACT also yielded significant findings (MD = 3.14, 95% CI: 2.15–4.13, *Z* = 6.22, *p* < 0.00001), although there is only one study among the three subgroup analyses, the *p* < 0.05 for each study indicates that the efficacy is evident.

#### 3.5.2 Subgroup analysis of MMSE scores

**Figure 10:**
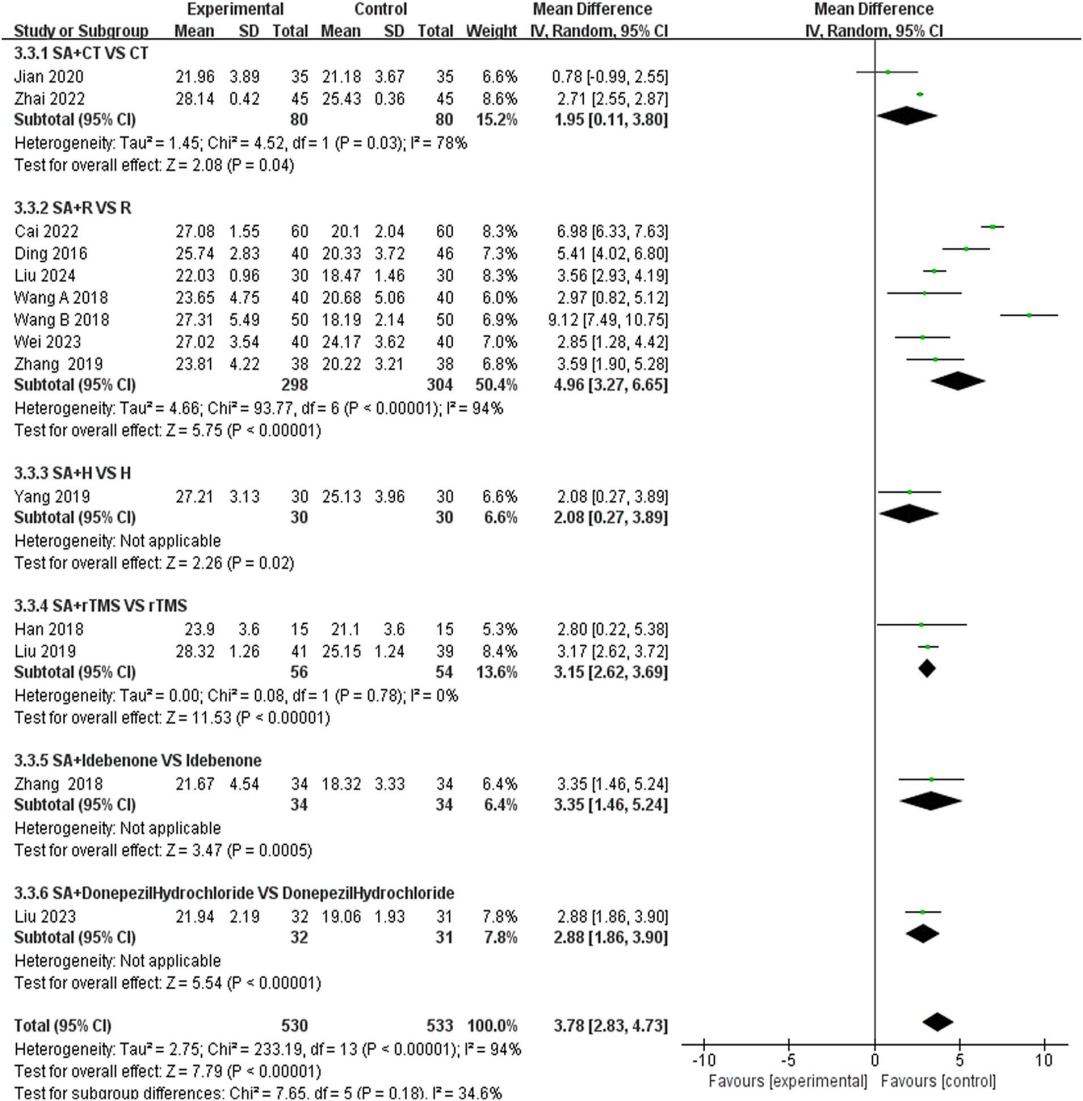
Forest plots of subgroup analysis of MMSE scores.

We further conducted a subgroup analysis of the 14 studies that recorded MMSE scores based on the type of treatment (Figure 10). The results indicated that the SA + CT vs. CT subgroup was significantly effective (MD = 1.95, 95% CI: 0.11–3.80, *I*^2^ = 78%, 2 studies, 160 participants). Similarly, the SA + R vs. R subgroup demonstrated significant effectiveness (MD = 4.96, 95% CI: 3.27–6.65, *I*^2^ = 94%, 7 studies, 602 participants). The SA + rTMS vs. rTMS subgroup also showed significant effectiveness (MD = 3.15, 95% CI: 2.62–3.69, *I*^2^ = 0%, 2 studies, 110 participants). Notably, the *I*^2^ was >75% in the SA + CT vs. CT and SA + R vs. R subgroups, suggesting high heterogeneity, which may compromise the reliability of these results. In contrast, the SA + rTMS vs. rTMS subgroup analysis, with an *I*^2^ = 0% (less than 50%), indicates low heterogeneity, suggesting that scalp acupuncture combined with rTMS treatment is more effective than rTMS alone in treating PSCI, rendering these results highly reliable. Furthermore, in the comparison of SA + donepezil hydrochloride vs. donepezil hydrochloride (MD = 2.88, 95% CI: 1.86–3.90, *Z* = 5.54, *p* < 0.00001), SA + H vs. H (MD = 2.08, 95% CI: 0.27–3.89, *Z* = 2.26, *p* < 0.02), SA + idebenone vs. idebenone (MD = 3.59, 95% CI: 1.90–5.28, *Z* = 4.17, *p* < 0.0001), there is only one study for each of the three subgroup analyses. Nonetheless, the *p*-values of each study were less than 0.05, indicating that the efficacy was evident.

#### 3.5.3 Subgroup analysis of LOTCA scores

**Figure 11:**
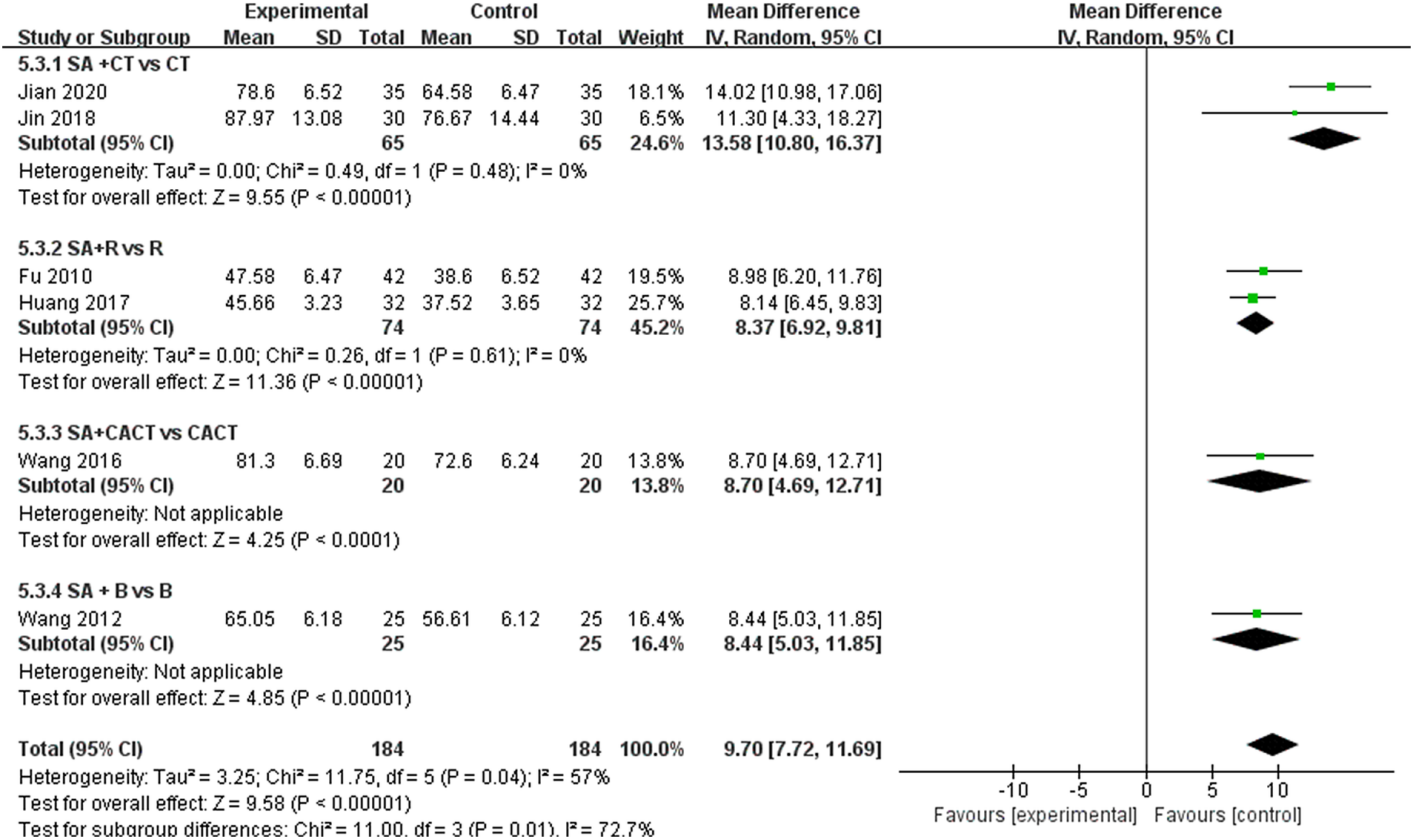
Forest plots of subgroup analysis of LOTCA scores.

We further conducted a subgroup analysis on the six studies that recorded LOTCA scores based on the type of treatment administered in the control group (Figure 11). The results indicated that the subgroup comparison of SA + CT vs. CT was significantly effective (MD = 13.58, 95% CI: 10.08–16.37, *I*^2^ = 0%, 2 studies, 130 participants). Similarly, the subgroup comparison of SA + R vs. R demonstrated significant effectiveness (MD = 8.37, 95% CI: 6.92–9.81, *I*^2^ = 0%, 2 studies, 148 participants). Since *I*^2^ = 0 < 50%, heterogeneity is considered low, suggesting that the results are reliable. Additionally, the comparison of SA + CACT vs. CACT (MD = 8.70, 95% CI: 4.69–12.71, *Z* = 4.25, *p* < 0.0001), SA + B vs. B (MD = 8.44, 95% CI: 5.03–11.85, *Z* = 4.85, *p* < 0.00001) Although each of the two subgroup analyses includes only one study, the *p* < 0.05 for each indicate that the efficacy is evident.

### 3.6 Publication bias and sensitive estimate

**Figure 12:**
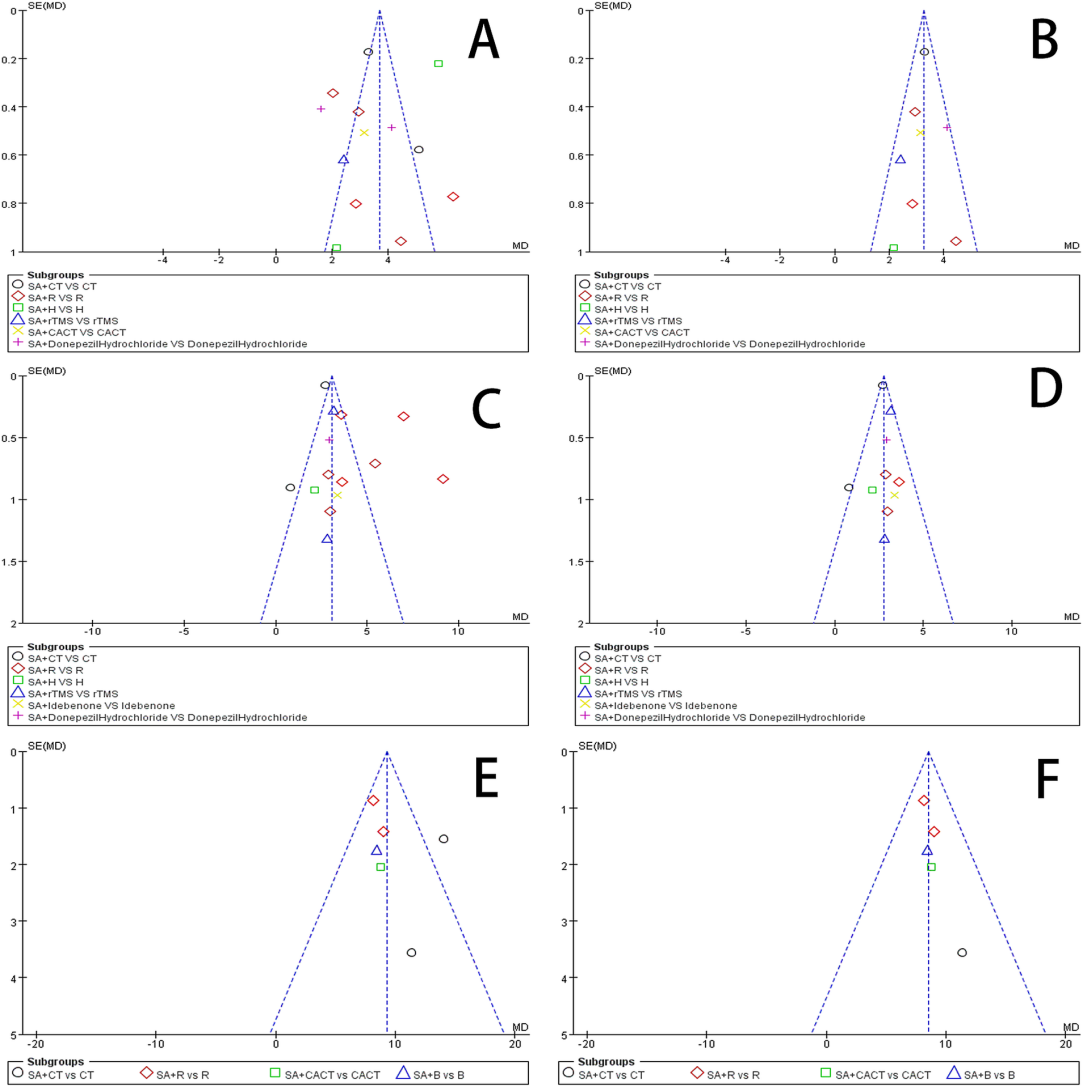
**(A)** Funnel plot of the MoCA scores. **(B)** Funnel plot of the MoCA scores after removing four studies. **(C)** Funnel plot of the MMSE scores. **(D)** Funnel plot of the MoCA scores after removing one from four studies. **(E)** Funnel plot of the LOTCA scores. **(F)** Funnel plot of the MoCA scores after removing one study.

An analysis of 13 studies documenting MoCA scores revealed that the funnel plot exhibited asymmetry, with 5 studies falling outside the 95% CI (Figure 12A). After excluding these 5 studies (Wang et al., 2017; Mao and Cheng, 2019; Chen A. Z. et al., 2020; Chen J. et al., 2020; Liu et al., 2024), we observed that *I*^2^ = 19 < 50%, indicating that the reliability of scalp acupuncture in improving cognitive dysfunction post-stroke is supported (MD = 3.25, 95% CI: 2.99–3.52, *I*^2^ = 19%, 8 studies, 643 participants) (Figure 5B). Nonetheless, the funnel plot still displayed slight asymmetry (Figure 12B). In the analysis of 14 studies that recorded MMSE scores, the funnel plot also showed asymmetry, with 4 studies falling outside the 95% CI (Figure 12C). Upon excluding these 4 studies (Ding and Zhang, 2016; Wang W. et al., 2018; Cai et al., 2022; Liu et al., 2024) (MD = 2.74, 95% CI: 2.59–2.89, *I*^2^ = 2%, 10 studies, 697 participants), we found that *I*^2^ = 2% < 50% (Figure 6B). At the same time, the funnel plot exhibited slight asymmetry (Figure 12D). Additionally, the analysis of 6 studies that recorded LOTCA scores indicated that the funnel plot was asymmetric, with 1 study falling outside the 95% CI (Figure 12E). After excluding this 1 study (Xiong et al., 2020) (MD = 8.50, 95% CI: 7.26–9.74, *I*^2^ = 0%, 5 studies, 298 participants), we determined that *I*^2^ = 0% < 50 (Figure 7B) and the funnel plot showed slight asymmetry (Figure 12F). A consistent conclusion was reached through the analysis of studies recording both MMSE and LOTCA scores.

Begg’s test and Egger’s test can detect the symmetry of the funnel plot to determine whether there is publication bias (Jin et al., 2015). The results of the Begg and Egger bias tests are presented in Table 3. The Egger bias tests for the prevalence of MoCA (*p* = 0.6590), MMSE (*p* = 0.1790), LOTCA (*p* = 0.4350), P300 latency (*p* = 0.7300), and P300 amplitude (*p* = 0.2590) indicated the absence of publication bias. However, the Egger bias test regarding the incidence rate of effective rate (*p* = 0.0100 < 0.05) revealed the presence of publication bias.

**Table 3:** Beeg test and Egger test for publication bias of outcome indicators reported in this study.

Outcome		Begg (*p*-value)		Egger (*p*-value)	
Effective rate		0.1665	>0.05	0.0100	<0.05
	After	0.7639	>0.05	0.1940	>0.05
MoCA		0.0019	<0.05	0.6590	>0.05
	After	0.0000	<0.05	0.5020	>0.05
MMSE		0.0031	<0.05	0.1790	>0.05
	After	0.1524	>0.05	0.8640	>0.05
LOTCA		0.4524	>0.05	0.4350	>0.05
	After	1.0000	>0.05	0.0610	>0.05
P300	(Latency)	0.4624	>0.05	0.7300	>0.05
	After	0.7341	>0.05	0.8210	>0.05
	(Amplitude)	1.0000	>0.05	0.2590	>0.05

### 3.7 Adverse events

One study (Liu Y. F. et al., 2023) observed that some patients did not adhere to the prescribed treatment and subsequently dropped out. The remaining 27 studies (Xie et al., 2007; Fu et al., 2010; Wang et al., 2012; Xie et al., 2012; Qin, 2015; Ding and Zhang, 2016; Wang, 2016; Huang et al., 2017; Wang et al., 2017; Du et al., 2018; Han et al., 2018; Wang W. et al., 2018; Wang Y. X. et al., 2018; Zhang and An, 2018; Yang L. et al., 2019; Liu et al., 2019; Mao and Cheng, 2019; Zhang et al., 2019; Chen A. Z. et al., 2020; Chen J. et al., 2020; Xiong et al., 2020; Zhang et al., 2020; Cai et al., 2022; Zhai et al., 2022; Li and Pan, 2023; Wei et al., 2023; Liu et al., 2024) did not report any adverse events. Consequently, we were unable to assess the safety of scalp acupuncture in the treatment of PSCI, which raises concerns about a potential risk of reporting bias.

## 4 Discussion

### 4.1 Main findings

The study encompassed 28 randomized controlled trials involving a total of 1,995 participants. It evaluated the efficacy of combined scalp acupuncture in treating PSCI by examining multiple outcome indicators, including the MoCA, MMSE, and LOTCA scale scores, as well as P300 latency and amplitude. The findings indicate that the combination of scalp acupuncture with CT, CACT, R, H, and rTMS is more effective than any single treatment modality. Notably, the evidence supporting the effectiveness of scalp acupuncture combined with R in improving P300 latency and amplitude in PSCI patients is considered reliable. Furthermore, there is moderate to high heterogeneity among the studies regarding MoCA, MMSE, and LOTCA scores, along with a moderate to high risk of bias. All included studies focused on the application of scalp acupuncture for patients in the acute stroke phase. Thus, this study concludes that combined scalp acupuncture is more effective in addressing cognitive impairment following acute stroke compared to individual therapies such as CT, CACT, R, H, and rTMS.

Subgroup analyses of studies examining the MoCA and MMSE scores across various intervention approaches revealed a consistent association with moderate to high risk. In contrast, studies focusing on LOTCA scores exhibited lower risks, suggesting that the findings from the subgroup analyses comparing SA + CT versus CT, SA + R versus R, SA + CACT, and SA + donepezil hydrochloride were reliable.

In the context of the scalp acupuncture treatment regimens examined in the studies included in this analysis, the majority utilized the International SA method, with the additional incorporation of Yu SA and Fang SA. The most frequently employed acupoints are the mid-frontal line (MS1), mid-parietal line (MS5), anterior parietotemporal oblique line (MS6), posterior parietotemporal oblique line (MS7), anterior temporal line (MS10), and posterior temporal line (MS11). Other notable acupoints include the Baihui point (DU20), Sishencong point (EX-HN1), Qianding (GV21), and Shenting (GV24). This suggests that SA treatment primarily targets the frontal, temporal, and parietal regions, which correspond to the frontal lobes, temporal lobes, and parietal lobes—areas closely associated with the brain’s learning, memory, executive functions, and visuospatial/structural abilities. However, the underlying mechanisms of action remain to be elucidated in future studies. The most common treatment duration is 30 min per session, with a typical operational frequency of 200 sessions per minute. This clinical treatment protocol may serve as a guide for future clinical applications.

Resting-state functional magnetic resonance imaging (resting-state fMRI) is a technology employed to investigate the treatment mechanisms of PSCI, observing changes in brain function by analyzing changes in resting blood oxygen level signals. This technology primarily evaluates three indicators: amplitude of low-frequency fluctuations (ALFF), regional homogeneity (ReHo), and functional connectivity (Zhang et al., 2021). Studies have confirmed that scalp acupuncture treatment can enhance ALFF values in various regions of the brain, including the hippocampus, cingulate gyrus, splenium cortex, right inferior temporal gyrus, left middle occipital gyrus, left superior occipital gyrus, right superior parietal gyrus, prelimbic cortex, and sensory cortex (Wen et al., 2018; Han et al., 2024). The effectiveness of this treatment is associated with an increase in spontaneous neuronal activity in these areas (Han et al., 2024), which may facilitate the recovery of overall cognitive function, executive abilities, attention, and speech expression. ReHo is a technique utilized for processing local brain functional activity data, with its values closely linked to cognitive functions (Veselinovic et al., 2022). Following PSCI, alterations in functional brain activity are observed. Research indicates that scalp acupuncture can positively influence ReHo values in the brain regions of PSCI patients, particularly in the superior parietal gyrus, right inferior frontal gyrus, right inferior temporal gyrus, precuneus, lentiform nucleus, ventrolateral nucleus of the thalamus, and middle temporal gyrus. Conversely, negative activation was noted in the lentiform nucleus, ventrolateral thalamic nucleus, middle temporal gyrus, and parahippocampal gyrus (Han et al., 2024). These findings indicate that scalp acupuncture treatment may enhance the regeneration and repair of synapses in nerve cells, thereby facilitating the recovery of brain regions associated with cognitive functions in patients with PSCI. Furthermore, PSCI can lead to abnormal functional connections within the brain’s cognitive-related networks, resulting in alterations to the brain’s information integration processes (Xiong et al., 2022). Studies have confirmed that scalp acupuncture can influence the topological properties of brain functional network nodes in patients with PSCI (Wang, 2021), modulating brain networks by increasing functional connectivity between brain regions related to cognitive activities (Tan et al., 2017) and thereby improving the cognitive function of patients to a certain extent.

Diffusion tensor imaging (DTI) is a non-invasive technique that visualizes brain white matter fiber tracts and detects changes in brain white matter microstructure *in vivo* (Zhu et al., 2019; Li X. T. et al., 2022). Following PSCI, abnormal alterations in white matter fiber tracts occur, with fractional anisotropy (FA) values in the frontal lobe, hippocampus, and corpus callosum typically decreasing. This reduction suggests that the white matter fibers in these regions may have sustained damage or degeneration, thereby impacting the brain’s information integration processes (Yin et al., 2019). Research has demonstrated that scalp acupuncture can enhance the blood supply to white matter tracts in the brain and facilitate the repair of white matter fiber damage, consequently improving cognitive function in PSCI patients (Sun and Zhang, 2020). Additionally, scalp acupuncture may stimulate the release of neurotransmitters and neuropeptides by enhancing cerebral blood circulation, which can slow neuronal apoptosis, activate neural pathways, and further enhance cognitive function (Zhou et al., 2020). Furthermore, scalp acupuncture can improve the metabolic levels of brain functional tissues (Zhang et al., 2020), increase blood circulation, and promote the release of neurotransmitters and neuropeptides, thereby contributing to the reduction of neuronal cell apoptosis, activation of brain neural pathways, and enhancement of cognitive function.

In terms of basic research, a substantial number of studies have demonstrated that scalp acupuncture can influence the expression of tumor necrosis factor-α (TNF-α), interleukin-1β (IL-1β), and other cytokines associated with the inflammatory response in rats with middle cerebral artery occlusion (MCAO) (Li et al., 2020). This effect is specifically characterized by the down-regulation of these cytokines’ expression levels. Concurrently, scalp acupuncture treatment has been shown to increase the expression of brain-derived neurotrophic factor (BDNF) (Zheng et al., 2020) and plasticity-related proteins such as postsynaptic density protein 95 (PSD-95) in the hippocampus (Li et al., 2020). Additionally, scalp acupuncture can enhance mitophagy and reduce apoptosis levels (Liu et al., 2021). Through these mechanisms, scalp acupuncture treatment contributes to mitigating the cascade effects following a stroke, improving neuronal deficits, and fostering the repair of motor, cognitive, speech, and other neuronal functions. Despite this, there is still a lack of more specific research on SA treatment of PICS that is different from other sequelae after stroke and cognitive function in the elderly.

In summary, the potential mechanisms by which scalp acupuncture may treat PSCI can be categorized into several key aspects: (1) it may increase spontaneous neuronal activity in brain regions associated with cognitive functions and enhance functional connectivity between these areas; (2) it may promote the regeneration of synapses and repair of nerve cells; (3) it could enhance blood supply to white matter tracts in the brain, improve overall blood circulation, stimulate the release of neurotransmitters and neuropeptides, while also slowing neuronal apoptosis and elevating the metabolic levels of functional brain tissues; (4) it may down-regulate levels of inflammation-related factors and promote the expression of BDNF and proteins associated with neuroplasticity in the hippocampus; and (5) it may downregulate apoptosis levels and increase mitophagy.

### 4.2 Characteristics of outcome indicators

Appropriate assistive tools are critical for the study of cognitive impairment following a stroke. Currently, commonly used cognitive function evaluation tools include MMSE, MoCA, Hasegawa Dementia Scale (HDS-R), the Oxford Cognitive Screening (OCS), and LOTCA (Wang and Dong et al., 2021; Li L. et al., 2022). This study utilized the most widely adopted assessment tools, specifically the MMSE and MoCA scores, as outcome measures. The MMSE is particularly sensitive to cognitive domains such as memory and language, especially in the context of left hemisphere stroke, and demonstrates high sensitivity and specificity for diagnosing dementia. However, its sensitivity to mild cognitive impairment is relatively low (Mancuso et al., 2018; Milosevich et al., 2024). In contrast, the MoCA compensates for this limitation, exhibiting higher sensitivity and specificity in identifying mild cognitive impairment. Both assessments are suitable for less educated individuals but may not be appropriate for patients with post-stroke aphasia or neglect (Mancuso et al., 2018; Milosevich et al., 2024). Conversely, the LOTCA is frequently employed in clinical settings to assess non-verbal cognitive dysfunction in patients with aphasia (Almomani et al., 2018). These considerations underscore the importance of addressing the limitations associated with the MMSE and MoCA scales.

Event-related potentials (ERPs) are specific types of electrical signals in the brain (Kayser and Tenke, 2015). Research has demonstrated a significant correlation between components of event-related potentials that peak at approximately 300 ms and cognitive function, making them a valuable tool for assessing cognitive performance in the brain (Tyson-Carr et al., 2018). P300 can reflect the brain’s memory, attention, and mental processing speed through variations in amplitudes and latencies (Morrison et al., 2019). Consequently, the P300 component offers a quantitative, objective, and non-invasive electrophysiological testing method for future research on cognitive impairment following a stroke.

### 4.3 Quality of the evidence

The randomized controlled trials analyzed in this study indicate that most items in the ROB2 assessment are categorized as low risk. However, several studies demonstrate unclear risks related to “participant and personnel blinding bias,” “allocation concealment procedures,” and “outcome assessment blinding bias,” which could potentially skew study results toward a positive outcome. Therefore, the principle of allocation concealment should be strictly adhered to in future trial design and implementation. Given the specific nature of scalp acupuncture treatment, it is not feasible to blind participants and operators, which diminishes the overall quality of the study. Furthermore, sensitivity analyses conducted during the meta-analysis revealed that 11 studies (Xie et al., 2012; Ding and Zhang, 2016; Wang et al., 2017; Wang W. et al., 2018; Wang Y. X. et al., 2018; Mao and Cheng, 2019; Chen A. Z. et al., 2020; Chen J. et al., 2020; Xiong et al., 2020; Cai et al., 2022; Liu et al., 2024) exhibited high heterogeneity. This heterogeneity may arise from imbalances between the control and experimental groups, unclear randomization methods, selective reporting, small sample sizes, and a lack of control over participants, possibly influenced by variations in cultural backgrounds. To address these issues, future studies should aim to increase sample sizes and the number of research centers, as well as assess the educational levels of subjects to enhance research quality. Consequently, the included studies were rated as being at high risk of implementation bias.

## 5 Limitation

This study has several limitations that merit discussion. First, due to the inherent nature of scalp acupuncture, neither the operator nor the subjects can be blinded. In many studies, the research design must delineate the randomization method; consequently, the quality of the included studies could have been enhanced. Second, during the meta-analysis, various factors contributed to high heterogeneity in specific outcomes. These factors include individual differences, varying treatment options (such as operator skill, the subject’s disease course, observation duration, and acupuncture point selection), stroke type (ischemic versus hemorrhagic), as well as the subject’s education level and degree of cognitive function impairment. Third, the general acceptance of scalp acupuncture treatment varies among individuals, and all studies included in this analysis were conducted in China, with limited engagement from scholars in other countries. This geographical limitation significantly undermines the reliability of the meta-analysis results. Finally, meta-analyses typically involve the aggregation of results from multiple trials, which increases the likelihood of random errors. Such errors can profoundly influence the outcomes of meta-analyses (Wetterslev et al., 2017). Therefore, future research should focus on strategies to mitigate the impact of random errors on the results of meta-analyses.

## 6 Conclusion

Although the sample size and methodological quality of the 28 randomized controlled trials included in this study were not entirely satisfactory, we observed changes in outcome indicators—specifically, the total effective rate, MMSE score, MoCA score, P300 latency and amplitude, and LOTCA score—before and after treatment, suggesting that scalp acupuncture can improve PSCI. Furthermore, due to the high risk of bias (ROB) in the included trials and the very low quality of the evidence for assessing outcomes, the results of this study should be interpreted with caution. Future clinical research should employ high-quality randomized double-blind controlled trial designs, utilize multi-center studies with large sample sizes, conduct long-term efficacy evaluations, and apply scientifically sound methods. Additionally, the procedures for scalp acupuncture should be standardized and unified.

## Data Availability

The original contributions presented in the study are included in the article/Supplementary material, further inquiries can be directed to the corresponding authors.
