# Supplementary material for: Efficacy of combination scalp acupuncture for post-stroke cognitive impairment: a systematic review and meta-analysis
Source: Front Neurosci. 2024 Nov 29;18:1468331. doi: 10.3389/fnins.2024.1468331 (PMC11638195; doi:10.3389/fnins.2024.1468331)
Supplement: Supplementary file 1 [file Data_Sheet_1.zip › 1468331_SupMaterial/S2 Appendix.DOCX]

| Search strategy for PubMed database. | |
| --- | --- |
| #1 | ((((((scalp acupuncture[Title/Abstract]) OR (head acupuncture[Title/Abstract])) OR (cranial acupuncture[Title/Abstract])) OR (cephalic acupuncture[Title/Abstract])) OR (scalp electric acupuncture[Title/Abstract])) OR (scalp penetration acupuncture[Title/Abstract])) OR (needle the head[Title/Abstract]) |
| #2 | ((("Stroke"[Mesh] ) OR ((((((((((((((((((((((((ischemic stroke[Title/Abstract]) OR (Stroke, Ischemic[Title/Abstract])) OR (Ischaemic Stroke[Title/Abstract])) OR (Ischaemic Strokes[Title/Abstract])) OR (Stroke, Ischaemic[Title/Abstract])) OR (Cryptogenic Ischemic Stroke[Title/Abstract])) OR (Cryptogenic Ischemic Strokes[Title/Abstract])) OR (Ischemic Stroke, Cryptogenic[Title/Abstract])) OR (Stroke, Cryptogenic Ischemic[Title/Abstract])) OR (Cryptogenic Stroke[Title/Abstract])) OR (Cryptogenic Strokes[Title/Abstract])) OR (Stroke, Cryptogenic[Title/Abstract])) OR (Cryptogenic Embolism Stroke[Title/Abstract])) OR (Cryptogenic Embolism Strokes[Title/Abstract])) OR (Embolism Stroke, Cryptogenic[Title/Abstract])) OR (Stroke, Cryptogenic Embolism[Title/Abstract])) OR (Wake-up Stroke[Title/Abstract])) OR (Stroke, Wake-up[Title/Abstract])) OR (Wake up Stroke[Title/Abstract])) OR (Wake-up Strokes[Title/Abstract])) OR (Acute Ischemic Stroke[Title/Abstract])) OR (Acute Ischemic Strokes[Title/Abstract])) OR (Ischemic Stroke, Acute[Title/Abstract])) OR (Stroke, Acute Ischemic[Title/Abstract])))) OR (cerebral injury[Title/Abstract]) |
| #3 | (("Cognitive Dysfunction"[Mesh]) OR (((((((((((((((((((((((((Cognitive Dysfunctions[Title/Abstract]) OR (Dysfunction, Cognitive[Title/Abstract])) OR (Dysfunctions, Cognitive[Title/Abstract])) OR (Cognitive Impairments[Title/Abstract])) OR (Cognitive Impairment[Title/Abstract])) OR (Impairment, Cognitive[Title/Abstract])) OR (Impairments, Cognitive[Title/Abstract])) OR (Cognitive Disorder[Title/Abstract])) OR (Cognitive Disorders[Title/Abstract])) OR (Disorder, Cognitive[Title/Abstract])) OR (Disorders, Cognitive[Title/Abstract])) OR (Mild Cognitive Impairment[Title/Abstract])) OR (Cognitive Impairment, Mild[Title/Abstract])) OR (Cognitive Impairments, Mild[Title/Abstract])) OR (Impairment, Mild Cognitive[Title/Abstract])) OR (Impairments, Mild Cognitive[Title/Abstract])) OR (Mild Cognitive Impairments[Title/Abstract])) OR (Cognitive Decline[Title/Abstract])) OR (Cognitive Declines[Title/Abstract])) OR (Decline, Cognitive[Title/Abstract])) OR (Declines, Cognitive[Title/Abstract])) OR (Mental Deterioration[Title/Abstract])) OR (Deterioration, Mental[Title/Abstract])) OR (Deteriorations, Mental[Title/Abstract])) OR (Mental Deteriorations[Title/Abstract])) OR (cognitive defect[Title/Abstract])) |
| #4 | ((randomized controlled trial[Publication Type] OR randomized[Title/Abstract] OR placebo[Title/Abstract])) |
| #5 | #1 and #2 and #3 and #4 |
