# Supplementary material for: Efficacy of combination scalp acupuncture for post-stroke cognitive impairment: a systematic review and meta-analysis
Source: Front Neurosci. 2024 Nov 29;18:1468331. doi: 10.3389/fnins.2024.1468331 (PMC11638195; doi:10.3389/fnins.2024.1468331)
Supplement: Supplementary file 1 [file Data_Sheet_1.zip › 1468331_SupMaterial/S3 Appendix.DOCX]

**Identification of studies via databases and registers**

Literature obtained by searching databases (N=367)

Duplicate records removed (n =112 )

**Identification**

Exclude Meta-analysis, systematic review, animal experiments, and master and doctoral conference papers(N=129)

Literatures obtained after primary screening(N=255)

Literatures obtained after primary screening(N=126)

Non-matching literature after browsing titles and abstracts(N=84)

**Screening**

Article included after full text review(N=42)

Reports excluded:

1.Excluding the inability to obtain the original text(N=7)

2.Excluding literatures with inconsistent study content (N=2)

3.Different documents use the same data(N=5)

The final included literature(N=28)

**Included**

*Consider, if feasible to do so, reporting the number of records identified from each database or register searched (rather than the total number across all databases/registers).

**If automation tools were used, indicate how many records were excluded by a human and how many were excluded by automation tools.

*From:*  Page MJ, McKenzie JE, Bossuyt PM, Boutron I, Hoffmann TC, Mulrow CD, et al. The PRISMA 2020 statement: an updated guideline for reporting systematic reviews. BMJ 2021;372:n71. doi: 10.1136/bmj.n71

For more information, visit: <http://www.prisma-statement.org/>
